# Oral Manifestations and Dental Management Considerations of Lipoid Proteinosis: A Case Report and Review of Literature

**DOI:** 10.30476/DENTJODS.2021.89748.1435

**Published:** 2022-09

**Authors:** Fatemeh Jahanimoghadam, Jelveh Hasheminejad

**Affiliations:** 1 Social Determinants on Oral Health Research Center, Kerman University of Medical Sciences, Kerman Iran; 2 Pediatric Dentistry Resident, School of Dentistry, Kerman University of Medical Sciences, Kerman, Iran

**Keywords:** Acneiform scar, Hoarseness, Oral Manifestation, Lipoid proteinosis, Moniliform blepharosis

## Abstract

Lipoid proteinosis (LP) is a sporadic congenital metabolic disorder characterized by deposition of hyaline material in various organs. It has a very low prevalence rate of approximately 300 cases reported up to now. It has a vast spectrum of manifestations ranging from asymptomatic skin lesions to the rare but life-threatening laryngeal obstruction. The knowledge of the clinical features of the disease such as hoarseness of voice from infancy, mucocutaneous manifestations, moniliform blepharosis (multiple, beaded papules along the eyelash line) and dental anomalies such as hypoplasia or aplasia of teeth may help oral health care practitioners improve the quality of their patient’s life. This case report describes a typical 10-year-old boy who presented to the Department of Pediatric Dentistry, Faculty of Dentistry, University of Medical Sciences, Kerman, Iran with the typical recurrent skin and mucosal lesions, hoarseness, and blepharosis. In addition, he stated a gradual hearing loss, which is not reported as a common manifestation. Moreover, psychosocial issues regarding his appearance and quality of voice had led to absenteeism from school. A punch biopsy obtained from a lesion on his forearm revealed the characteristic histopathological view and directed to the diagnosis of lipoid proteinosis. Dental treatment was initiated with focus on preventive dentistry due to the restricted mouth opening, which was expected to get worse overtime. There is no definitive cure for this disease and the treatment is symptomatic in most cases. A proper workup can result in early diagnosis and management of dental complications, which are difficult to control due to restricted mouth opening, which may significantly affect the patient’s social life.

## Introduction

Lipoid proteinosis (LP) is a rare, autosomal recessive genetic disease first described by Siebenmann in 1908[ 1 ]. This condition is also known by other terms such as “Urbach–Weithe disease”“, lipoglycoproteinosis,” “lipid proteinosis,” and “hyalinosis cutis et mucosae”. It presents as a disease with a range of ophthalmic, cutaneous, neurologic, and oral manifestations, characterized by deposition of hyaline material in various organs [ 1 - 3 ]. Currently, the exact etiology of LP is unknown but studies indicate that a homozygous or compound heterozygous mutation in the extracellular matrix gene 1 (ECM1) on chromosome 1q21 is related to the condition. The mutated ECM1 gene generates abnormalities in the glycolipid or sphingolipid pathway, causing deposits of hyaline material in the mucous membranes of the upper aerodigestive tract and skin [ 4 - 6 ]. A review of literature revealed approximately 300 LP cases reported up to now [ 1 , 3 - 4 ]. LP occurs worldwide, but it is most commonly reported in North and South Africa especially in areas with higher rate of inbreeding marriages. No definitive age, sex, or race predilections have been reported [ 7 - 10 ]. However, a systematic literature review performed in 2019, with a focus on cases reported in the Middle East and North Africa reported the highest prevalence in Turkey. In this review, male to female ratio was stated to be 1:1.25, and a mean age of seventeen years was reported [ 11 ].

The characteristic histopathological findings are hyperkeratosis and pigmentary incontinence in the basal layer, periodic acid‐schiff (PAS)‐reactive hyaline deposits in the upper dermis, with localization around blood vessels and eccrine sweat glands, in particular [ 12 - 13 ]. 

The first clinical manifestation is hoarseness, often recognized immediately after birth. This scrawniness in the crying voice of an infant is due to hyaline deposition in the laryngeal mucosa [ 3 - 4 ]. Other mucocutaneous manifestations usually occur in the first two years of life, they present as waxy papules, blisters, acneiform scarring and a progressive thickening of the skin and oral mucosa [ 3 - 4 , 8 ]. A characteristic feature of this disease is moniliform blepharosis presented as multiple, beaded papules along the eyelash line [ 14 ]. Other manifestations in some LP patients are reported as alopecia, nail dystrophy, dental anomalies such as hypoplasia or aplasia of teeth, epilepsy, memory impairment, typical intracranial calcifications, lung involvements, and insulin resistance [ 5 , 7 - 8 , 15 ]. 

The clinical findings along with the histopathological examination confirm the diagnosis of lipoid proteinosis. The disease is slowly progressive and commonly has a benign course. However, the extensive skin lesions and hoarseness can lead to severe psychosocial issues. In this report, we present an LP patient with a gradual hearing loss in addition to the more common manifestations.

## Case Presentation

A 10-year-old boy was referred to the Department of Pediatric Dentistry, Faculty of Dentistry, University of Medical Sciences, Kerman, Iran in the year 2020 with a chief complaint of a burning sensation in the mouth while eating, recurring itchy skin lesions, and dysphagia. Swallowing was very difficult for him and he needed to drink water frequently in order to be able to swallow. He also stated a gradual hearing loss, which was later verified by an ENT specialist. Moreover, psychosocial issues regarding his appearance and quality of voice had made him leave the school.

**Figure 1:**
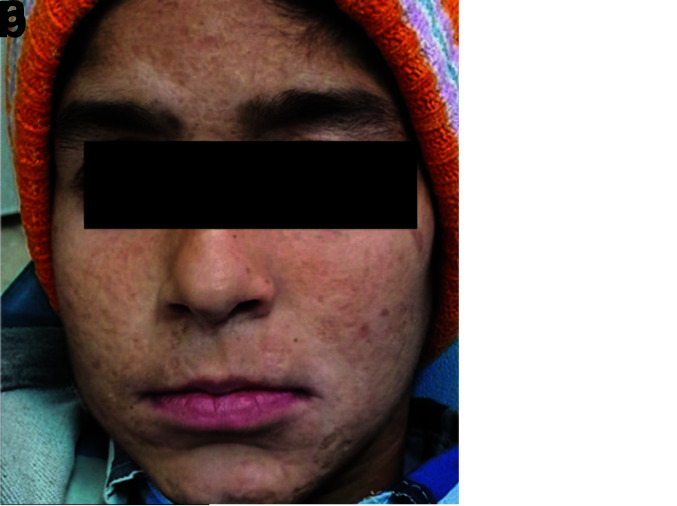
**a:** Frontal view demonstrating acneiform scars and blisters on the face and **b:** acneiform scars and blisters on forearm

His medical history revealed hoarseness of voice since infancy followed by typical skin lesions when he was around 3 years old. Skin lesions were evident as puss or blood-filled blisters on the entire body especially face and dorsum of hands. The history shows that the lesions appear, heal, and reappear over a period. The lesions were healed by scaring and for that reason, the body parts slowly started to become stiff. Mild pain was reported in the initial stages of larger skin lesions. Multiple brownish macules and papules were present on dorsum of hands and arms (Figure 1). His voice quality also deteriorated with age and became low and squeaky. He had no history of seizures or visual disturbances. He also mentioned respiratory distress on exertion. To now, he had received no medical treatment for the clinical manifestations mentioned above.

Family history revealed that the child was born of consanguineous marriage (first cousins) in a family with a low socioeconomic status. His five-year-old brother was also affected with similar skin lesions but hoarseness of voice was not much evident in him. None of the other family members, including his 3-year-old sister and his parents, was affected; however, his nephew was mentioned to have the same disease.

**Figure 2:**
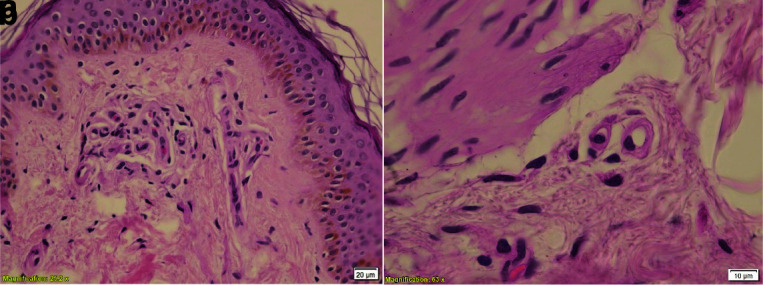
**a:** Histopathologic view revealing slight keratosis and mild acanthosis of pigmented epidermis, **b:** Thickened, homogenized, hyalinized walls of vessels

A punch biopsy including epidermis, dermis, and subcutaneous tissues was obtained from a lesion on his forearm, which revealed slight keratosis and mild acanthosis of pigmented epidermis. In addition, the papillary and upper small vessels presented thickened, homogenized, hyalinized walls (Figure 2). This characteristic histopathological view along with the typical clinical features, lead to the diagnosis of LP. 

Studies reveal an association between bilateral amygdalae calcifications and LP [ 6 , 14 ]. However, this was not evident in our case. On general examination, patient was cooperative and well oriented to time and place.

**Figure 3:**
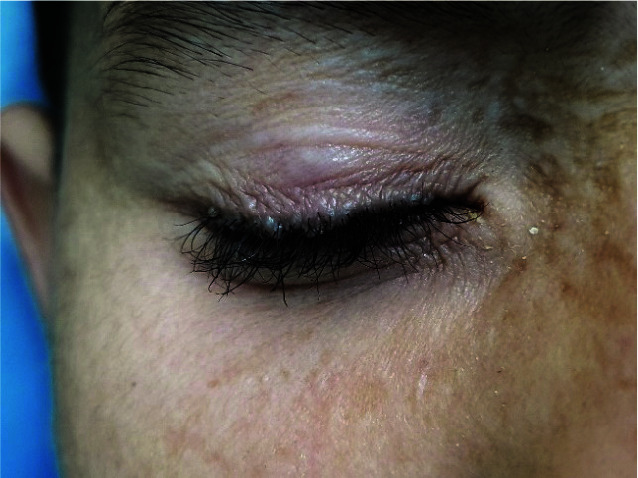
Moniliform blepharosis (multiple, beaded papules along the eyelash line)

**Figure 4:**
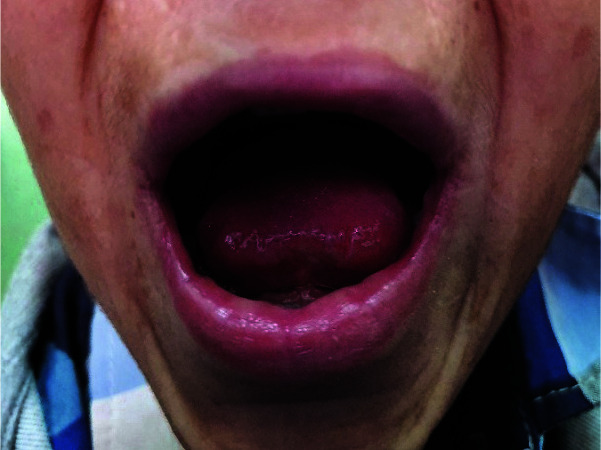
Restricted mouth opening

On extra oral examination of head and neck, apart from the acneiform skin lesions, the characteristic beaded papules, also known as moniliform blepharosis were evident on the thickened margins of upper eyelids (Figure 3). On intra oral examination, mouth opening was restricted, with maximal interincisal opening limited to 20mm (Figure 4).

**Figure 5:**
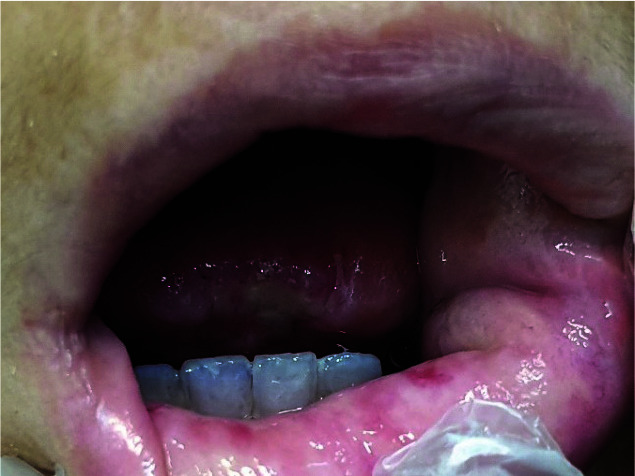
Severe ulceration of tip of the tongue, firm nodular buccal and lingual mucosa

**Figure 6:**
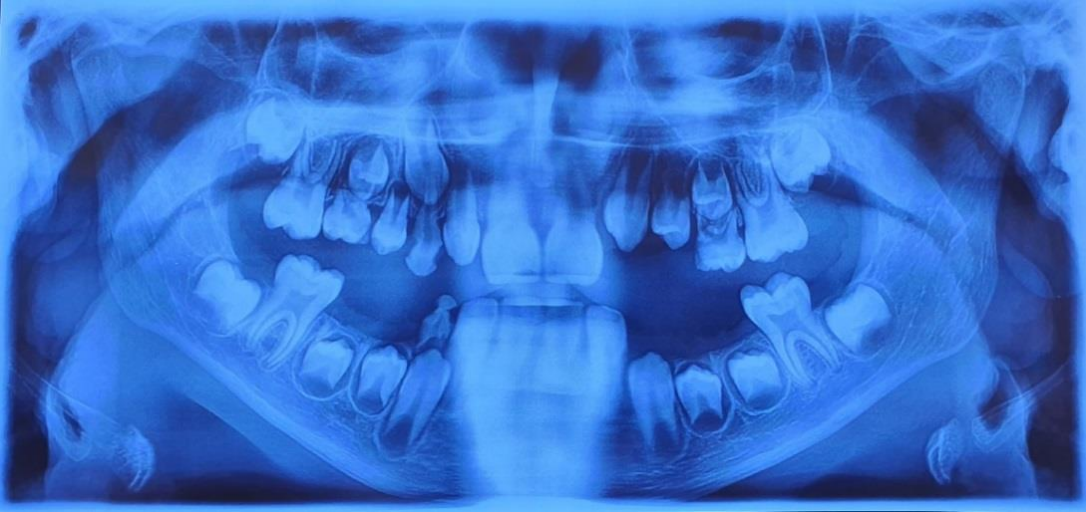
Panoramic radiograph reveals missing of upper left lateral incisor and no other pathological lesion

However, on examination of the temporomandibular joints, no abnormality was present. The joints were auscultated during mandibular motion and no crepitus or clicking was present. Palpation of the masticatory muscles revealed no tenderness. This suggested the reduced skin elasticity as a major reason for the restricted mouth opening. An enlarged, yellowish tongue with irregular pearly white infiltrations was another feature noted. His tongue had a hard consistency and the lingual frenum was thick, short and indurated, which limited its movement. He could not protrude his tongue beyond his lip margins. Severe ulceration of tip of the tongue was noted as a result of sensitivity to mild trauma. On palpation, both buccal and labial mucosa were firm and multiple nodules were felt (Figure 5). Oral mucosa was dry and hyposalivation was evident. The panoramic radiograph as the radiological examination presented missing of upper left lateral incisor but no pathological lesion (Figure 6). 

Dental treatment was initiated with focus on preventive dentistry due to the restricted mouth opening which was expected to get worse overtime. Dental treatments included sealing of pits and fissures of the first permanent molars with flowable composite. After consulting an orthodontist, the upper and lower deciduous canines were extracted due to extensive caries, also the upper second deciduous molar was extracted in order to facilitate mesial movement of first permanent molar and achieve a class II occlusion without spacing. Follow up appointments were scheduled to assess tooth movement and ultimately align and reshape the permanent canine as a lateral incisor. Four lower incisors and three upper incisors were caries free. The remaining deciduous teeth had been extracted earlier due to severe pain. As for the dry mouth, some practices were advised to alleviate symptoms, such as staying hydrated by ensuring adequate fluid intake by using a straw to drink, chewing sugar-free gum, or sucking on hard sugar-free candy to increase saliva production and sipping on liquids throughout the day to keep the mouth moist. If these practices failed to alleviate symptoms of dry mouth, the patient was advised to swish a small amount of liquid saliva substitute for 30 seconds (exactly as directed on the label), then spit it out. This procedure should be repeated 3 to 5 times per day. This also can be done when the mouth feels dry and uncomfortable. Furthermore, a proper oral hygiene regimen was initiated. Patient was referred to a maxillofacial surgeon for the treatment and management of ankyloglossia. He was also referred to a dermatologist for skin lesions and an ENT specialist for the hoarseness of voice and assessment of the stated hearing loss. 

## Discussion

LP is a slowly progressing condition in which materials resembling lipids and proteins accumulate in various organs, thus the disease is named LP [ 9 ]. 

LP results from mutations in ECM1. Exons 6 and 7 are the most common sites for ECM1 mutations in LP. More than 50 mutations have been reported on the 1q21 chromosome in the ECM1 gene up to now, which lead to deposition of hyaline-like material in the skin, mucosa, and internal organs [ 1 , 3 ]. Clinically, it appears that the more severe mucocutaneous LP phenotype is related to mutations outside exon 7 [ 5 ].

LP must be differentiated from a few conditions including erythropoietic protoporphyria. Erythropoietic protoporphyria presents with similar skin lesions but they are confined to sun exposed areas. Oral involvement is not seen in erythropoietic protoporphyria and most patients report photosensitivity. Furthermore, in erythropoietic protoporphyria deposition of PAS-positive material never occur around sweat glands. Apart from erythropoietic protoporphyria, which is the most closely resembling condition to LP, histologically LP should be differentiated from amyloidosis and xanthomas [ 2 - 3 ].

Bilateral oval calcification in the region of the medial temporal lobes is a characteristic feature in LP, with a reported incidence of 52% of cases [ 6 ]. Epilepsy, mental dysfunction, and neuropsychiatric abnormalities have been reported when intracranial calcifications were evident [ 1 ]. These findings were more evident in patients with longer disease duration. Considering the critical role of temporal lobes in the process of hearing, speech, memory, and emotions, the gradual hearing loss stated in our patient may be due to early stages of temporal lobe involvement, which was not now evident in his MRI. This can emphasize the importance of periodic follow-ups in order to prevent further progression if possible [ 16 ]. To now, hearing loss has not been reported as a manifestation of LP but our case reveals the possibility of a correlation between hearing disorders and LP.

Patients affected by LP present various clinical manifestations, for instance, Chakrabarti *et al.* [ 17 ] reported a patient with LP and dwarfism. No specific cause was obtained to explain the reason for this short stature but it was suggested that defective osteoblasts, which are similar to fibroblasts biologically, might be related to this uncommon presentation [ 17 ]. Poyrazoğlu *et al.* [ 5 ] also reported an LP patient with short stature; however, his osteoblastic activity markers were normal. Uchida *et al.* [ 18 ] report a case of calcinosis cutis occurring in LP. Calcium deposits were seen in the dermis. There was no clinical evidence of such calcifications in our case. Bazopoulou-Kyrkanidou *et al.* [ 19 ] described a 66-year-old man with LP who presented with generalized gingival hyperplasia due to diffuse deposition of hyaline-like material. Moreover, cases have been reported in which LP has been seen in siblings born to nonconsanguineous marriage, although parental consanguinity is a feature documented in most of the cases [ 19 ]. Kachewar and Kulkarni [ 9 ] reported a case of LP with an atypical finding in skull radiograph. In their case, cranial calcification had an irregular form and was evident in the posterior cranial fossa, which was not reported earlier. Furthermore, studies revealed other complications such as corneal opacities or secondary glaucoma due to infiltration in the trabeculae [ 19 ], possible gastrointestinal bleeding in case of small bowel involvement [ 9 ], involvement of salivary glands, which causes hyposalivation leading to poor oral hygiene [ 19 ]. 

There is no definitive cure for this disease. In most cases, the treatment is symptomatic. Several medications have been considered for the treatment of skin lesions including dimethyl sulfoxide, etretinate and its active metabolite acitretin, intralesional heparin, and D-penicillamine. However, none of these medicines has shown consistently good results. Dermabrasion may be recommended to patients whose skin lesions would not improve by the routine oral medications. . As for the eyelid and aero digestive tract lesions, carbon dioxide laser is an option, which should also be considered. Moreover, removing airway lesions by using micro laryngoscopy has shown improvement in airway and sound quality [ 3 ]. Anticonvulsants are prescribed for patients with frequent seizures [ 3 , 9 ]. Although this condition is rarely life threatening but death has been reported due to respiratory obstruction [ 3 , 6 ].

An informed consent was taken from the patients’ legal guardians (parents) for publishing the photographs and medical history.

## Conclusion

Lipoid proteinosis is a disease, which can disturb many aspects of the patient’s life and be quite exasperating. A proper workup can result in early diagnosis and timely management, which may subsequently decrease the complications such as the life-threatening laryngeal obstruction, the extensive skin lesions that can significantly affect the patient’s social life, and dental complications, which are difficult to manage due to restricted mouth opening. Hence, it is imperative for oral health care practitioners to have profound knowledge to diagnose and provide appropriate counseling and treatment to such patients.

## Acknowledgment

The authors wish to thank the patient and his parents for their assistance all through the study.

## Conflict of Interest

The authors declare that they have no conflict of interest.
